# Serum Levels of FGF-21 Are Increased in Coronary Heart Disease Patients and Are Independently Associated with Adverse Lipid Profile

**DOI:** 10.1371/journal.pone.0015534

**Published:** 2010-12-29

**Authors:** Zhuofeng Lin, Zhen Wu, Xiaojing Yin, Yanlong Liu, Xinxin Yan, Shaoqiang Lin, Jian Xiao, Xiaojie Wang, Wenke Feng, Xiaokun Li

**Affiliations:** 1 School of Pharmacy, Wenzhou Medical College, Wenzhou, China; 2 Department of Endocrinology, the 3rd Affiliated Hospital, Sun Yat-Sen University, Guangzhou, China; 3 Department of Cardiovascular Diseases, the 6th Affiliated Hospital, Sun Yat-Sen University, Guangzhou, China; 4 School of Medicine, University of Louisville, Louisville, Kentucky, United States of America; 5 Bioreator Engineering Research Center, Minister of Education, Jilin Agricultural University, Changchun, China; 6 The Key Laboratory of Pathobiology, Ministry of Education, Norman Bethune College of Medicine, Jilin University, Changchun, China; University of Hong Kong, China

## Abstract

**Background:**

Fibroblast growth factor 21 (FGF-21) is a metabolic regulator with multiple beneficial effects on glucose homeostasis and lipid metabolism in animal models. The relationship between plasma levels of FGF-21 and coronary heart disease (CHD) in unknown.

**Methodology/Principal Findings:**

This study aimed to investigate the correlation of serum FGF-21 levels and lipid metabolism in the patients with coronary heart disease. We performed a logistic regression analysis of the relation between serum levels of FGF-21 and CHD patients with and without diabetes and hypertension. This study was conducted in the Departments of Endocrinology and Cardiovascular Diseases at two University Hospitals. Participants consisted of one hundred and thirty-five patients who have been diagnosed to have CHD and sixty-one control subjects. Serum FGF-21 level and levels of fasting blood glucose; triglyceride; apolipoprotein B100; HOMA-IR; insulin; total cholesterol; HDL-cholesterol; LDL-cholesterol; and C-reactive protein were measured. We found that median serum FGF-21 levels were significantly higher in CHD than that of control subjects (P<0.0001). Serum FGF-21 levels in CHD patients with diabetes, hypertension, or both were higher than that of patients without these comorbidities. Serum FGF-21 levels correlated positively with triglycerides, fasting blood glucose, apolipoprotein B100, insulin and HOMA-IR but negatively with HDL-C and apolipoprotein A1 after adjusting for BMI, diabetes and hypertension. Logistic regression analysis demonstrated that FGF-21 showed an independent association with triglyceride and apolipoprotein A1.

**Conclusions/Significance:**

High levels of FGF-21 are associated with adverse lipid profiles in CHD patients. The paradoxical increase of serum FGF-21 in CHD patients may indicate a compensatory response or resistance to FGF-21.

## Introduction

The fibroblast growth factor (FGF) family is composed of 22 members with a wide range of biological functions, including cell growth, development, angiogenesis, and wound healing [1]–[7]. FGF-21 is a member of the endocrine FGF subfamily, which also includes FGF23, human FGF19, and its mouse homolog FGF15 [8]–[12]. In mice, FGF-21 is expressed predominantly in the liver and stimulates glucose uptake through the induction of GLUT1 in adipocytes [8]. In vivo, treatment with FGF-21 resulted in amelioration of glucose and lipid parameters in both murine and nonhuman primate models of diabetes and obesity [13], [14]. Furthermore, FGF-21-treated animals exhibited increased energy expenditure, fat utilization, lipid excretion, reduced hepatosteatosis, and ameliorated glycemia in diet-induced obese and ob/ob mice [13]. On the other hand, adenovirus-mediated down regulation of FGF-21 in the liver led to the development of fatty liver, dyslipidemia, and reduced serum ketones due to the altered expression of key genes involved in hepatic lipid and ketone metabolism [15]–[17]. Taken together, these findings demonstrate an important role of FGF-21 as a hepatic hormone in the regulation of lipid metabolism and also suggest that FGF-21 exhibits the therapeutic characteristics necessary for an effective treatment of obesity and fatty liver disease.

Recent human studies indicate that increased circulating levels of FGF-21 are found in obese individuals and subjects with metabolic syndrome or type 2 diabetes [18], [19] and are closely associated with obesity [18], [20] and renal dysfunction in chronic hemodialysis [21]. However, no data have been published about the relationship between this growth factor and coronary heart disease (CHD).

To explore the physiological and pathological characteristics of FGF-21 in patients with CHD, we measured the serum concentrations of FGF-21 in 196 Chinese subjects and analyzed its association with a cluster of metabolic parameters. Unexpectedly, our data demonstrated that serum FGF-21 levels are significantly increased in CHD individuals and are independently associated with adverse lipid metabolism.

## Methods

### Participant Selection and Sample Collection

Based on the clinical diagnostic criteria shown as following, individuals suffered from angina pectoris or myocardial infarctions were enrolled into this study as CHD subjects. Myocardial infarction was diagnosed on the basis of electrocardiogram findings, elevation of serum enzymes, and chest discomfort consistent with myocardial infarction. Angina pectoris was defined as recurrent chest discomfort related to exercise or excitement that lasted up to 15 minutes that was responsive to rest or nitroglycerin and together with objective evidence of myocardial ischemia (≥1 mm of ST-segment depression or ≥2 mm of T-wave inversion on an electrocardiogram at rest or inducible ischemia on exertion or pharmacologic stress testing). 135 CHD patients (67 males/68 females) were recruited in this study. 61 subjects (31 males and 30 females) served as controls. All control subjects were selected based on the results of physician's questionnaire and clinical biochemical examination. The CHD patients who received medication within at least one year for lipid lowering, anti-hypertension and diabetic treatment were excluded. Written informed consent was given to all patients and control individuals, and all the procedures were approved by the Ethics Committee of Wenzhou Medical College.

### Anthropometric and biochemical measurements

All subjects were assessed after overnight fasting for at least 10 h. The details of anthropometric measurements (height, weight, BMI, waist circumference, and blood pressure) and the methods for assay of biochemical variables (fasting glucose, insulin, total cholesterol, triglycerides, LDL and HDL cholesterol and lipoprotein (a) (Lp(a)) were obtained as reported previously [22]. Insulin resistance was estimated using homeostasis model assessment index–insulin resistance (HOMA-IR) [23], [24]. Serum levels of FGF-21 (Biovendor, Modrice, Czech Republic) and C-reactive protein (CRP, R&D, USA)were determined with commercially available enzyme-linked immunosorbent assays according to the manufacturers' instructions.

After an overnight fasting, blood samples were taken. Serum insulin was measured with a two-site chemiluminescent enzyme immunometric assay using Immulite automated analyzer (Diagnostic Products, Los Angeles, CA). Fasting blood glucose, total cholesterol, HDL-c, apolipoprotein A1 (ApoA1), apolipoprotein B100 (ApoB100), LDL-c, and triglycerides were measured by standard laboratory methods in a certified clinical examination laboratory.

### Statistical analysis

All analyses were performed with Statistical Package for Social Sciences version 13.0 (SPSS, Chicago, IL) similarly as described by Zhang et al[20]. Briefly, normally distributed data were expressed as mean±SD. Data that were not normally distributed, as determined using Kolmogorox-Smirnov test, were logarithmically transformed before analysis and expressed as median with interquartile range. Student's unpaired t test was used for comparison between two groups. Pearson's correlation was used as appropriate for comparisons between groups, and multiple testing was corrected using Bonferroni correction. Multiple stepwise regression analysis was used to examine the association of serum FGF-21 and other parameters. The variables correlated significantly with serum FGF-21 (after Bonferroni correction for multiple testing) were selected to enter into stepwise regression. In all statistical tests, P values <0.05 were considered significant.z

## Results

### Serum FGF-21 levels are increased in CHD patients

**Figure 1:**
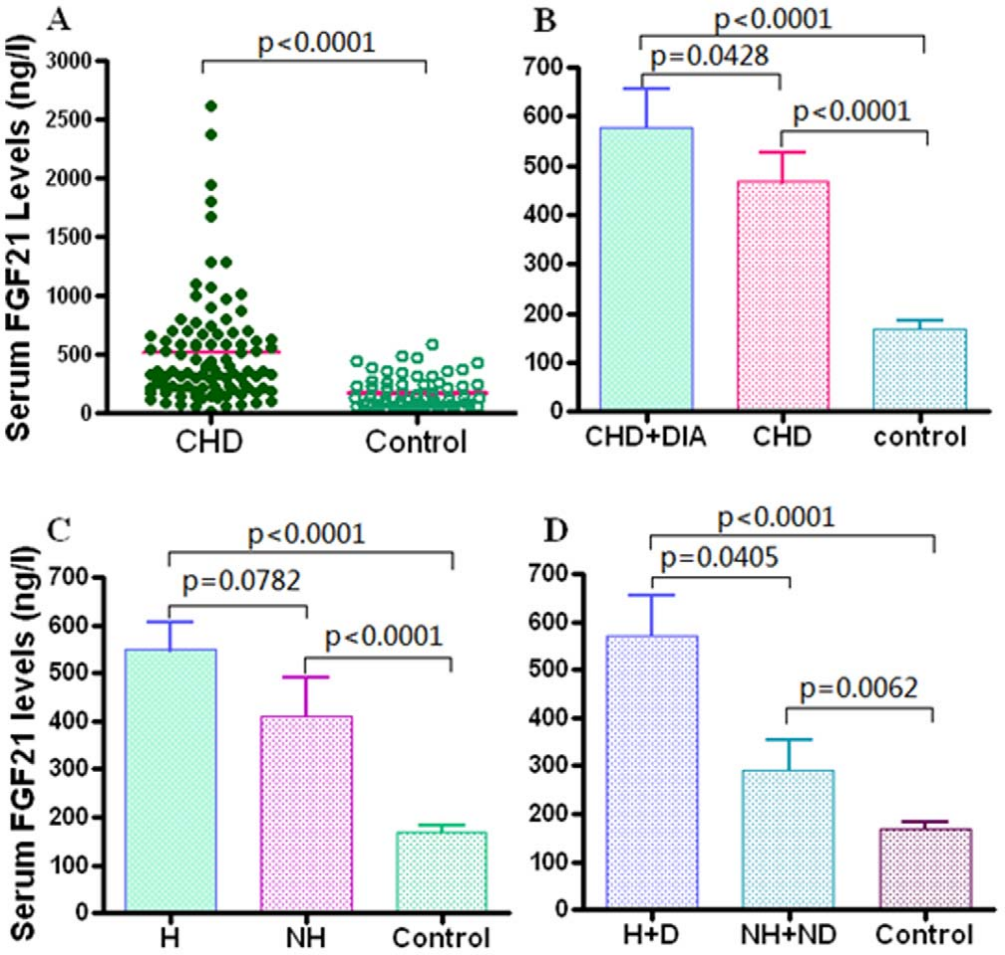
Serum FGF-21 concentrations in subjects with CHD and normal controls. A, Serum FGF-21 levels of 135 CHD patients and 61 control subjects. B, Serum FGF-21 levels of CHD patients with diabetes (CHD+DIA, n = 61) and CHD patients without diabetes (CHD, n = 74). C, Serum FGF-21 levels of CHD patients with hypertension (H, n = 97) and CHD patients without hypertension (NH, n = 38). D, Serum FGF-21 levels of CHD patients with diabetes and hypertension (H+D, n = 45) and CHD patients without diabetes and hypertension (NH+ND, n = 21).

Biochemical and clinical characteristics of the CHD patient and healthy groups are summarized in Table 1. Serum FGF-21 levels ranged from 11.63 to 2614.9 ng/l in 196 subjects, and there were no sex differences in serum FGF-21 levels (men [n = 94], median 415.3 ng/l [interquartile range 58.5, 250.1] vs. women [n = 92], 361.0 ng/l [79.4, 218.3], P = 0.252). Interestingly, median circulating FGF-21 level was 3-fold higher in CHD patients (513.5±47.5 ng/l) compared with control subjects (167.9±15.6 ng/l) (P<0.0001) after adjusted for body mass index (BMI) (Table 1 and Figure 1A). On other hand, CHD subjects with diabetes (530.0 ng/l, [interquartile rang 75.6–1941.3], n = 61) had significantly higher serum FGF-21 levels (P = 0.034) than those of CHD subjects without diabetes (317.2 ng/l, [59.1–680.5], n = 74) (Figure 1B), consistent with the finding by Chen et al [25]. No significant difference in serum FGF-21 levels was observed between CHD patients with hypertension (462.2 ng/l, [59.1–2614.9], n = 97) and CHD patients without hypertension (428.2 ng/l, [77.8–1069.2] n = 38), despite an elevated trend in these CHD patients with hypertension (P = 0.0718, Figure 1C). Furthermore, serum FGF-21 levels in CHD patients with both diabetes and hypertension (502.3 ng/l, [157.6–2614.9], n = 45) were significantly higher than that of CHD patients without diabetes and hypertension (410.5 ng/l, [77.8–1069.2], n = 21, p = 0.026, Figure 1D). Logistic regression analysis showed that serum FGF21 level was independently associated with the prevalence of CHD (p = 0.004).

**Table 1:** Clinical and biochemical characteristics of CHD and control groups.

	Variables	Controls(n = 61)	CHD(n = 135)	P value
Anthropometric	Age(years)	68.6±10.8	69±5.8	-
	Gender, male(%)	31(50.8%)	67(49.6)	-
	BMI(kg/m^2^)	22.3±2.4	24.6±3.1	-
	Type 2 diabetes mellitus (%)	-	62(45.9)	-
	Hypertension (%)	-	63(46.7)	-
	Systolic pressure (mmHg)	123±21	135±61	0.009
	Diastolic pressure (mmHg)	73±12	74±23	NS
Lipid metabolism	Total Cholesterol (mmol/L)	4.37±0.62	4.77±1.02	0.036
	HDL-cholesterol(mmol/L)	1.51±0.39	1.07 ±0.27	<0.001
	LDL-cholesterol (mmol/L)	2.63±0.47	2.95±0.94	0.009
	Triglyceride (mmol/L)	1.15±0.44	1.94±1.59	<0.001
	ApoA1(g/L)	1.49±0.12	1.19±0.27	<0.001
	ApoB100(g/L)	0.76±0.11	0.95±0.33	<0.001
	Lip A (mg/L)	204.5±105.6	212.1±138.6	NS
Glucose metabolism	Fasting glucose (mmol/L)	5.08±0.51	6.31±2.18	<0.001
	Fasting insulin (mIU/L)	5.44±3.24	9.95±8.59	0.001
	HOMA-IR	1.22±0.10	2.88±1.31	<0.001
	HOMA-IS	73.96±54.5	101.8±89.9	0.035
Inflammation	Hs-CRP(mg/l)	1.16±0.83	6.67±2.71	<0.001
	FGF-21(ng/L)*	131.0(70.4 to 249.5)	362.5(210.6 to 661.9)	<0.001

Data are means ± SD or *median (interquartile range).

NS, not significant.

### Association of serum FGF-21 levels with adverse lipid metabolism and insulin resistance

**Figure 2:**
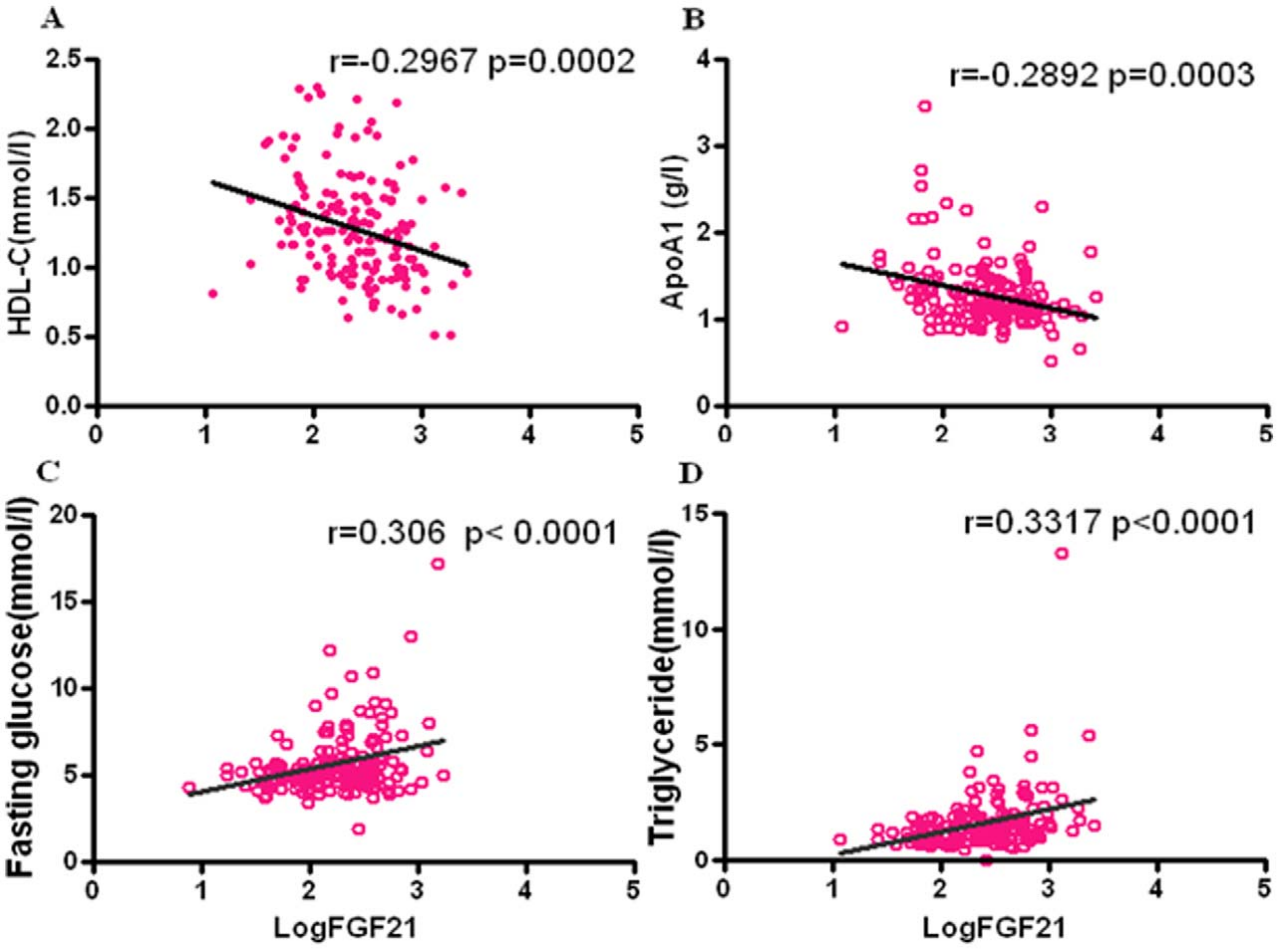
Correlation of serum FGF-21 levels with adverse lipid metabolism and insulin resistance. Regression analysis of serum levels of FGF-21 (log transformed) with HDL-cholesterol (A), Fasting glucose (B), Triglyceride (C) and Apolipoprotein AI (D) in 196 subjects.

As shown in Table 2 and Figure 2, a significant positive association of serum FGF-21 levels with triglyceride and apolipoprotein B100 (r = 0.365, P<0.001; r = 0.227, P = 0.002, respectively) after adjustment for BMI was observed. Serum FGF-21 levels correlated negatively with HDL- c and apolipoprotein A levels (r = −0.313, P<0.001; r = −0.338, P<0.00,1 respectively) after adjustment for BMI. However, the associations of serum FGF-21 with TC and LDL-c were not significant in our research subjects. In addition, serum FGF-21 levels correlated with fasting glucose, fasting insulin as well as HOMA-IR after adjustment for BMI, but no significant correlation was found between serum FGF-21 levels and HOMA-IS.

**Table 2:** Correlations of serum FGF-21* levels with anthropometric parameters and biochemical indexes.

Variables	Serum FGF-21		Serum FGF-21#	
	r	p	r	P
Age	−.004	NS	-	-
Gender	−.089	NS	-	-
BMI	0.239	0.001	-	-
Fasting Glucose	0.347	<0.001	0.305	<0.001
Fasting Insulin	0.209	0.012	0.192	0.027
Total Cholesterol	0.119	NS	0.144	NS
HDL-Cholesterol	−0.313	<0.001	−0.285	<0.001
LDL-Cholesterol	0.049	NS	0.035	NS
Triglyceride	0.365	<0.001	0.312	<0.001
Apolipoprotein A1	−0.338	<0.001	−0.310	<0.001
Apolipoprotein B100	0.227	0.006	0.196	0.012
Hs-CRP	0.102	NS	0.092	NS
Lp(a)	−0.154	NS	−0.122	NS
HOMA-IS	−.018	NS	−0.012	NS
HOMA-IR	0.255	0.002	0.232	0.006

*Log transformed before analysis.

# adjusted by BMI, diabetes and hypertension.

NS: not significant.

### Serum FGF-21 levels are independently associated with ApoA1, TG and fasting glucose

To determine whether serum FGF-21 was independently associated with anthropometric parameters and cardiovascular risk factors, stepwise logistic regression analysis involving all the parameters with significant correlations with serum FGF-21 was performed (Table 3). Stepwise logistic regression analysis revealed that ApoA1 (β coefficient −0.304, 95% CI −0.500 to –0.173, P<0.001), fasting glucose (β coefficient 0.219, 95% CI 0.013 to 0.087, P = 0.008) and triglyceride (β coefficient 0.213, 95% CI 0.016–0.124, P = 0.011) independently associated with serum FGF-21 after adjustment for BMI, diabetes and hypertension. However, all other parameters including total cholesterol, HDL-cholesterol, LDL-cholesterol, ApoB100, insulin and HOMA-IR were excluded during regression analysis.

**Table 3:** Multiple stepwise regression analysis showing variables independently associated with the serum level of FGF-21.

Independent Variables	Standardized β	B (95% CI)	t	P
Triglyceride	0.213	0.070 (0.016 to 0.124)	2.575	0.011
Apolipoprotein A1	−0.304	−0.343 (−0.500 to −0.173)	−4.117	<0.001
Fasted blood glucose	0.219	0.0503(0.013 to 0.087)	2.676	0.008

The analyses also included BMI, Total Cholesterol, HDL-cholesterol, LDL-cholesterol, ApoB100, insulin, HOMA-IS and HOMA-IR, which were all excluded in the final model.

## Discussion

Our primary aim was to observe the clinical manifestation of FGF-21 in conjunction with physiological and pathological aspects in CHD patients. Our results suggest that FGF-21 is correlated with CHD as supported by two novel findings. First, we demonstrate that in addition to fasting glucose, both apolipoprotein A1 and triglyceride are significantly associated with circulating FGF-21 in our research subjects independent of BMI, HDL cholesterol and insulin in multivariate analysis. Furthermore, we show that FGF-21 levels are 3-fold higher in CHD individuals with severely impaired cardiovascular systems.

While numerous data have been published in animal model, little is known about FGF-21 in human subjects, particularly for patients with heart diseases. Previous studies indicated that circulating FGF-21 is significantly higher in obese populations with increased cardiovascular risk [20]. Here we showed, for the first time, the pathological manifestation of FGF-21 in CHD individuals.

Serum FGF-21 levels were found to closely related to lipid metabolism. A recent study has already demonstrated that serum triglycerides and total cholesterol were independently associated with FGF-21 [19]. In this study, we found that apolipoprotein A1 was negatively correlated with FGF-21 concentration. Apolipoprotein A1 is the major protein component of the high-density lipoprotein (HDL) particles, which promotes cholesterol efflux from tissues to the liver for excretion. Studies have shown inverse associations between apoA1 levels and CHD [26], [27], and enhancing ApoA1 is preventive against CHD. FGF21 treatment provides beneficial changes in some lipoproteins and cardiovascular risk factors in rodents and nonhuman primates. However, it does not change circulating ApoA1 levels [14]. The correlation between FGF21 and ApoA1 in CHD deserves further studies.

We have also showed that diabetes and hypertension have additional effects on FGF-21 levels in CHD patients, which is supported by the fact that serum FGF-21 levels in CHD individuals with diabetes, hypertension, or both were higher than in the CHD individuals without these complications. However, diabetes and hypertension are unlikely to be a primary factor for the increment of FGF-21 in CHD patients, since CHD patients without diabetes and hypertension had significantly higher FGF-21 levels than normal controls.

The physiological and pathological significance of increasing serum FGF-21 levels in cardiovascular disease remains to be elucidated. As shown in previous studies [20], serum FGF-21 levels are significantly higher in obese population with increased cardiovascular risk characterized by the evidence of metabolic syndrome in the obese subjects. Because FGF-21 is an adipokine with glucose-lowering effects [8], [13], [28]–[30], it was speculated that the paradoxical increase of this protein in populations with risk of cardiovascular disease is a compensatory mechanism to counteract metabolic stress. Furthermore, FGF-21 resistance might be found in obesity and in renal failure, leading to compensatory upregulation of this adipokine [20], [21]. This gives us a speculative clue that may suggest that the mechanism of increased FGF-21 levels in cardiovascular disease is similar to obesity-associated resistance to insulin. Further studies are needed to elucidate the precise mechanism by which CHD subjects elevate circulation FGF-21 levels and reveal the role of increased FGF-21 levels in CHD.

There are several limitations in this study. Rather small collective of patients enrolled in this study is a major limitation of the study. A large sample size may be needed for more comprehensive investigation. In addition, our study did not address the cause-effect relationship between FGF-21 and the onset and development of CHD. Further studies are warranted to determine whether elevated serum FGF-21 is causally related to dyslipidemia or is a compensatory response to CHD.

In summary, this study provides clinical evidence revealing that serum concentrations of FGF-21 are increased in CHD subjects. Together with previous findings [20], our data suggest that serum concentrations of FGF21 in humans are not related to insulin secretion, but rather to lipid metabolism. The consistent increase in FGF21 seen in human CHD patients raises the intriguing possibility that FGF21 could be a biomarker for CHD.
